# Evaluation of Reproductive Histology Response of Adult *Fasciola hepatica* in Goats Vaccinated with Cathepsin L Phage-Exposed Mimotopes

**DOI:** 10.3390/ijms25137225

**Published:** 2024-06-29

**Authors:** Abel Villa-Mancera, Javier Maldonado-Hidalgo, Manuel Robles-Robles, Jaime Olivares-Pérez, Agustín Olmedo-Juárez, José Rodríguez-Castillo, Noemi Pérez-Mendoza, Fernando Utrera-Quintana, José Pérez, Samuel Ortega-Vargas

**Affiliations:** 1 Facultad de Medicina Veterinaria y Zootecnia, Benemérita Universidad Autónoma de Puebla, Tecamachalco Puebla 75460, Mexico; mvzjemh98@gmail.com (J.M.-H.); manuel.roblesr@correo.buap.mx (M.R.-R.); jose.rodriguez@correo.buap.mx (J.R.-C.); noemi.perezmen@correo.buap.mx (N.P.-M.); fernando.utrera@correo.buap.mx (F.U.-Q.); samuel.ortega@correo.buap.mx (S.O.-V.); 2 Unidad Académica de Medicina Veterinaria y Zootecnia, Universidad Autónoma de Guerrero, Ciudad Altamirano 39640, Mexico; olivaares@hotmail.com; 3 Centro Nacional de Investigación Disciplinaria en Salud Animal e Inocuidad (CENID SAI-INIFAP), Carretera Federal Cuernavaca-Cuautla No. 8534, Colonia Progreso, Jiutepec 62550, Mexico; aolmedoj@gmail.com; 4 Departamento de Anatomía y Anatomía Patológica Comparadas y Toxicología, Unidad de Excelencia ENZOEM, Universidad de Córdoba, 14014 Córdoba, Spain; an1pearj@uco.es

**Keywords:** *Fasciola hepatica*, reproductive histology, egg production, goats, phage display, vaccine development, cathepsin L mimotopes, ovary, vitellaria, Mehlis’ gland, testis

## Abstract

Fasciolosis, a globally re-emerging zoonotic disease, is mostly caused by the parasitic infection with *Fasciola hepatica*, often known as the liver fluke. This disease has a considerable impact on livestock productivity. This study aimed to evaluate the fluke burdens and faecal egg counts in goats that were administered phage clones of cathepsin L mimotopes and then infected with *F. hepatica* metacercariae. Additionally, the impact of vaccination on the histology of the reproductive system, specifically related to egg generation in adult parasites, was examined. A total of twenty-four goats, which were raised in sheds, were divided into four groups consisting of six animals each. These groups were randomly assigned. The goats were then subjected to two rounds of vaccination. Each vaccination involved the administration of 1 × 10^13^ phage particles containing specific mimotopes for cathepsin L2 (group 1: PPIRNGK), cathepsin L1 (group 2: DPWWLKQ), and cathepsin L1 (group 3: SGTFLFS). The immunisations were carried out on weeks 0 and 4, and the Quil A adjuvant was used in combination with the mimotopes. The control group was administered phosphate-buffered saline (PBS) (group 4). At week 6, all groups were orally infected with 200 metacercariae of *F. hepatica*. At week 22 following the initial immunisation, the subjects were euthanised, and adult *F. hepatica* specimens were retrieved from the bile ducts and liver tissue, and subsequently quantified. The specimens underwent whole-mount histology for the examination of the reproductive system, including the testis, ovary, vitellaria, Mehlis’ gland, and uterus. The mean fluke burdens following the challenge were seen to decrease by 50.4%, 62.2%, and 75.3% (*p* < 0.05) in goats that received vaccinations containing cathepsin L2 PPIRNGK, cathepsin L1 DPWWLKQ, and cathepsin L1 SGTFLFS, respectively. Animals that received vaccination exhibited a significant reduction in the production of parasite eggs. The levels of IgG1 and IgG2 isotypes in vaccinated goats were significantly higher than in the control group, indicating that protection is associated with the induction of a mixed Th1/Th2 immune response. The administration of cathepsin L to goats exhibits a modest level of efficacy in inducing histological impairment in the reproductive organs of liver flukes, resulting in a reduction in egg output.

## 1. Introduction

Fasciolosis is a zoonotic disease that is transmitted through food and is caused by the parasitic trematode *Fasciola hepatica*. This pathogen has the ability to infect several mammalian species, with a particular affinity for animals and humans. The global population facing the risk of infection from liver fluke is estimated to be at least 180 million individuals. Additionally, the number of individuals currently infected with liver fluke worldwide is estimated to range between 35 and 72 million, with indications of a potential upward trend [1,2]. The global economic losses caused by fasciolosis in livestock production are estimated to exceed USD 3 billion annually [3,4]. In the specific case of Mexican cattle, the estimated losses amount to USD 119 million [5]. The prevalence rates of fasciolosis in cattle throughout various American countries and regions vary from 3.0% to 66.7%. The caprine population has the greatest incidence rates, ranging from 24.5% to 100%, suggesting a significant susceptibility of goats to liver fluke infection [6,7]. However, there is a lack of evidence supporting the development of acquired resistance to this infection [8]. A high prevalence of caprine fasciolosis has been reported in the northwest region of Mexico, as indicated by the results of indirect enzyme-linked immunosorbent assay (ELISA) and faecal examinations, with prevalence rates of 43.0% and 24.5%, respectively [9]. In the Mixteca region, high prevalence rates were observed using coproantigen and serum IgG1 ELISA tests, with rates of 77.20% and 73.46%, respectively [10]. The increasing concern regarding the presence of chemical residues in milk and meat, the emergence of drug-resistant strains, and the substantial expenses associated with treatment have generated a heightened focus on the advancement of a vaccine that possesses both safety and environmental sustainability. Multiple antigens, including native and recombinant proteins, derived from *F. hepatica* have been identified [11,12]. One such antigen is cathepsin L, a protease that is highly expressed in the parasite. Cathepsin L can be classified into five distinct clades according to their sequence identities: clades of cathepsin L2 and L3 can cleave native collagen, facilitating migration through host tissues, while cathepsins L1, L2, and L3 degrade fibrinogen and fibrin (cathepsins L1 and L2) [13,14,15,16]. Clades of cathepsins L1, L2, and L5 can digest the haemoglobin and cathepsins L1 and L2 participate in the modulation of the host’s immune response by cleaving immunoglobulin in the hinge region [17,18]. The administration of recombinant cathepsin L to goats has been documented to result in a reduction in fluke load by 38.7% and 39.13% [19,20].

Each adult fluke produces up to 20,000 eggs per day, as hermaphrodite, mature *F. hepatica* possess two highly branched testes and a single dendritic ovary, alongside well-developed vitelline glands. The generation of spermatozoa and egg components represents the primary energy-intensive processes in mature parasites [21,22]. The process of egg formation in *F. hepatica* encompasses various organs, such as the ovary, responsible for oocyte production, and the vitellaria, which generate the vitelline cells (about 20–30 per egg) [22,23].

Phage display is a powerful tool and cost-effective for the straightforward identification of distinct peptide sequences that exhibit unique recognition by the immune system in the context of viral, bacterial, or parasitic diseases [24,25]. The technique of phage display is utilised to generate a diverse repertoire of peptides that are presented on the surface proteins of filamentous phages, specifically pIII or pVIII [26]. This method has demonstrated efficacy in the field of parasitology, particularly in the selection of epitopes or mimotopes using antibodies found in antisera obtained from animals that were previously immunised [27,28]. The objective of this study was to investigate the impact of immunisation with cathepsin L mimotopes in goats infected with *F. hepatica* metacercariae on fluke burden, faecal egg production, serum levels of antibodies, and the reproductive system of the parasite. The testis, ovary, vitellaria, Mehlis gland, and uterus were examined to identify the tissues most affected by immunisation with phage clones.

## 2. Results

### 2.1. Parasite Burden and Faecal Egg Count

The mean fluke burden per animal was expressed as mean ± SD was 47.2 ± 19.2 for group 1 (cathepsin L2 PPIRNGK); 36.0 ± 6.2 for group 2 (cathepsin L1 DPWWLKQ); 23.5 ± 7.6 for group 3 (cathepsin L1 SGTFLFS); and 95.2 ± 22.2 for the unimmunised control group. Hence, within the control group, the mean number of *F. hepatica* metacercariae worm burden resulting from the initial inoculum of 200 was 95.2, thereby giving an implantation rate of 47.6%. In the vaccinated groups, the mean values for the implantation rate were 23.6% for group 1, 18.0% for group 2, and 11.8% for group 3. Table 1 shows the reduction in parasites observed in the vaccinated groups compared to the control group. The animals that were administered the PPIRNGK sequence (group 1, cathepsin L2) exhibited a significant decrease of 50.4% in fluke burden, while group 2 (DPWWLKQ, cathepsin L1) showed a reduction of 62.2% in comparison to the control group that was infected. Group 3 (SGTFLFS, cathepsin L1) had the highest reduction in parasite burden, with 75.3% (*p* < 0.05).

In Table 1, the mean total faecal egg count (FEC) is shown for all groups that were infected with *F. hepatica* metacercariae. The control group consisting of unimmunised goats and the group of vaccinated animals in group 3 exhibited the highest and lowest egg outputs at week 22, respectively. The mean EPG (eggs per gramme) of vaccinated goats were determined at 58.6 ± 37.5 for group 1, 64.0 ± 25.4 for group 2, 35.4 ± 14.5 for group 3, and 122.0 ± 44.0 for the control group. The egg production at week 22 exhibited a significant reduction of 52.0% in group 1, 47.5% in group 2, and 71.0% in group 3 (*p* < 0.05) when compared to the control group that was not immunised.

### 2.2. Humoral Responses Induced by Vaccination 

**Figure 1:**
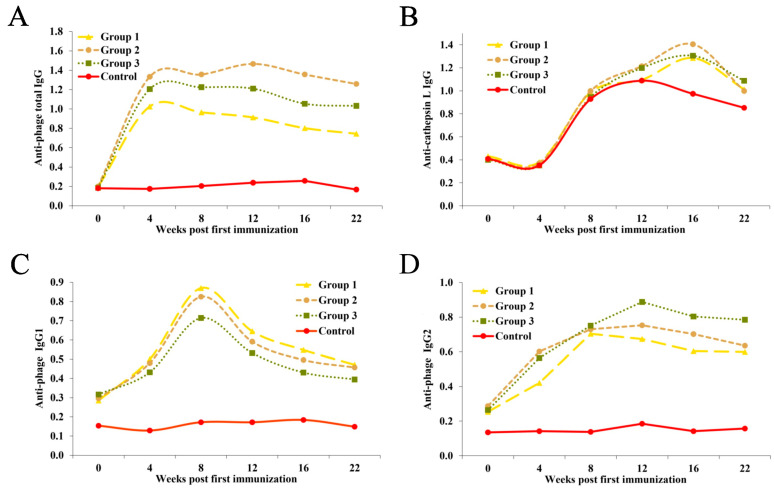
Total IgG levels against cathepsin L mimotopes (**A**) and cathepsin L native (**B**) in sera from goats infected with 200 *F. hepatica* metacercariae at week 6. Goats were given two immunisations at 0 and 4 weeks with 1 × 10^13^ pfu and Quil A adjuvant. IgG1 (**C**) and IgG2 (**D**) reactivity against selected phage clones in sera from control and vaccinated animals. Results are the mean of the six animals run in triplicate wells. Group 1: CL2 (PPIRNGK); group 2: CL1 (DPWWLKQ); group 3: CL1 (SGTFLFS); control group: PBS. CL: cathepsin L.

In vaccinated goats, a high humoral response against the homologous phages was observed, with a peak of 4 weeks after the first immunisation, and a slight decrease until the end of the study (Figure 1A). Group 2 with adjuvant Quil A showed a second increase in antibody levels after the second vaccination at week 12. The response was stronger of groups 2 (CL1: DPWWLKQ) and 3 (CL1: SGTFLFS) with adjuvant Quil A, followed by that of group 1 (CL1: PPIRNG), and lowest in the control group. The humoral responses to phage clones of all groups were examined by indirect ELISA using anti-cathepsin L native as antigens (Figure 1B). After the first injection, all goats showed a slight decrease in the production of antibody response until week 4. In addition, an increase was observed in the absorbance of the three vaccinated groups with adjuvant within 2 weeks following the challenge (at week 8), with a peak at 16 weeks after the first immunisation and a decline thereafter. The analysis of IgG subclasses produced by vaccination with phage clones of cathepsins L1/L2 mimotopes was analysed by indirect ELISA (Figure 1C,D). The results indicate that the serum IgG1 and IgG2 response was highest in goats immunised with phage clones compared to the control group. The phage-specific IgG1 levels peaked by week 8 and then decreased gradually until week 22. The mean absorbance values of vaccinated animals (groups 2 and 3) showed an elevation of IgG2 after the first immunisation until week 12 (except for group 1, where the peak was reached by week 8), which gradually decreased until week 22 when animals were euthanised. Using a nonparametric Spearman’s correlation test, goats vaccinated with cathepsin L1 (group 2: DPWWLKQ and cathepsin L1 (group 3: SGTFLFS), the fluke burden was significantly correlated with total IgG (r = −0.84, *p* < 0.05 and r = −0.77, *p* < 0.05, respectively).

### 2.3. Reproductive Histology of F. hepatica in Goats Immunised with Filamentous Phage Clones

#### 2.3.1. Testes of Parasites in Goats Vaccinated with Cathepsin L and Unvaccinated Goats

**Figure 2:**
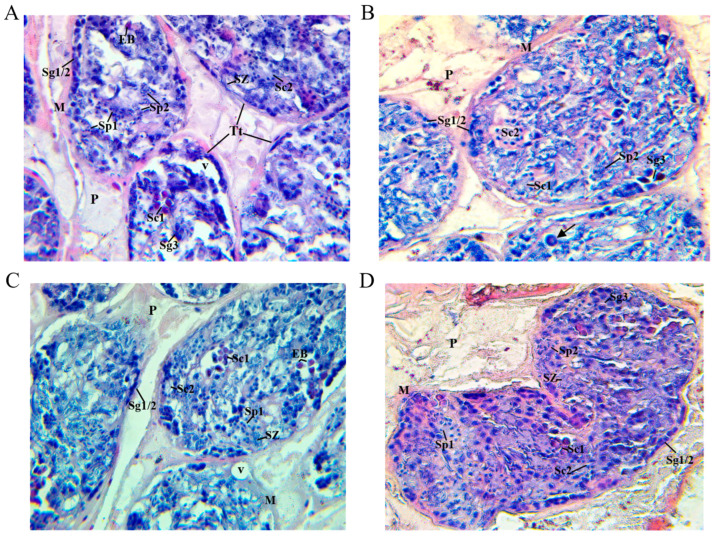
Light micrograph at 400× magnification of *Fasciola hepatica* seminiferous tubule tissue stained by H&E technique of vaccinated goats from group 1 (**A**), group 2 (**B**), group 3 (**C**), and unvaccinated control group 4 (**D**). (**A**) Each tubule is located in the parenchyma **(P)**, which is bounded by an extensive layer of smooth muscle **(M)**. The seminiferous tubules **(Tt)** show a small number of primary and secondary spermatogonia **(Sg1/2)** at the periphery of the tubules, while tertiary spermatogonia **(Sg3)** are found in the central zone of the tubules, as well as primary spermatocytes **(Sc1)** with multiple intense nuclei at the periphery of the eosinophilic cytoplasm, giving rise to eosinophilic bodies **(EB)**. Secondary spermatocytes **(Sc2)**, spermatids **(Sp1)**, and elongated spermatid nuclei **(Sp2)** are present in the central areas of the tubule. Small numbers of mature spermatozoa **(SZ)** are observed. (**B**) Each tubule is located in the parenchyma **(P)**, which is bounded by an extensive layer of smooth muscle **(M)**. Small numbers of primary and secondary spermatogonia **(Sg1/2)** and tertiary spermatogonia **(Sg3)** are seen, while primary spermatocytes **(Sc1)**, secondary spermatocytes **(Sc2)**, and spermatids with elongated nuclei **(Sp2)** are also present. Multiple nuclei of primary spermatocytes at the periphery of the eosinophilic cytoplasm (arrow). (**C**) Each tubule is located in the parenchyma **(P)**, which is bounded by an extensive layer of smooth muscle **(M)**. A small number of primary and secondary spermatogonia **(Sg1/2)** and tertiary spermatogonia **(Sg3)** are seen, as well as a moderate number of primary spermatocytes **(Sc1)** with eosinophilic bodies **(EB)**, also known as apoptotic cells. Vacuolation **(V)** is observed in the periphery and centre of the seminiferous tubules **(Tt)**, while secondary spermatocytes **(Sc2)**, spermatids **(Sp1),** and elongation of the spermatid nucleus **(Sp2)** are identified. (**D**) Each tubule is located in parenchymal tissue **(P)** and is bordered by an extensive layer of smooth muscle **(M)**. There are normal numbers of primary and secondary spermatogonia **(Sg1/2)** and apparently normal tertiary spermatogonia **(Sg3)**. Primary spermatocytes **(Sc)** with eosinophilic cytoplasm and a number of secondary spermatocytes **(Sc2)** are present, as are spermatids **(Sp1)** and elongated spermatid nuclei **(Sp2)**. A large number of mature spermatozoa **(Sz)** are observed.

Histological sections stained with haematoxylin and eosin (H&E) from trematodes recovered from the four groups showed spermatozoa and cell production in most of the testicular tubules, representing all stages of the spermatogenesis and spermiogenesis process [29,30,31]. The histological features of the seminiferous tubules of trematodes from groups 1–3 (vaccinated goats) and control group 4 (unvaccinated goats) are shown in Figure 2. The peripheral zones of the seminiferous tubules of groups 1–4 were occupied by primary and secondary spermatogonia, and the cells found in the centre of each tubule were identified as tertiary spermatogonia, clusters of primary spermatocytes (8 cells), secondary spermatocytes (8 cells), secondary spermatogonia (16 cells), secondary spermatocytes (16 cells), and spermatids (32 cells) [21]. The numbers of primary and secondary spermatogonia in the transverse sections of the seminiferous tubules were 32.5 ± 6.6, 31.1 ± 5.1, 16.4 ± 4.3, and 51.3 ± 8.6 in groups 1–3 and 4, respectively, with a significant reduction in the vaccinated groups (*p* < 0.01), particularly in group 3, with respect to the unvaccinated group. In addition, multiple irregular or fragmented condensed nuclei directed towards the periphery of the eosinophilic cytoplasm were observed in primary spermatocytes, resulting in a minimal amount of eosinophilic bodies (EBs). 

Trematodes from group 3 also showed severe alterations in the cells in the seminiferous tubules, with a high number of primary spermatocytes undergoing condensation and fragmentation of the nucleus with eosinophilic cytoplasm, as well as vacuolation, represented by a cell-free space located in the centre and periphery of the seminiferous tubules (Figure 2C). In the unvaccinated control group 4, injected with sterile PBS, there was a normal number of primary, secondary, and tertiary spermatogonia, as well as primary and secondary spermatocytes and spermatids, and spermatid nucleus elongation, corresponding to normal seminiferous tissue (Figure 2D).

#### 2.3.2. *Fasciola hepatica* Ovarian Tubules 

**Figure 3:**
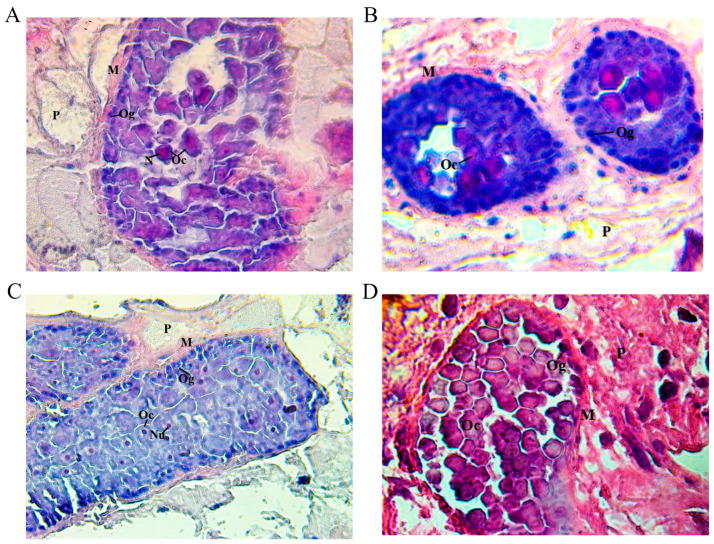
Light micrograph at 400× magnification of *Fasciola hepatica* ovarian tubule tissue stained by H&E technique of vaccinated goats from group 1 (**A**), group 2 (**B**), group 3 (**C**), and unvaccinated control group 4 (**D**). (**A**) The dendritic ovarian tubule is embedded in the parenchyma **(P)**, which is surrounded by a thick layer of smooth muscle **(M)**, with oogonia **(Og)** at the periphery of the tubule. Oocytes **(Oc)** are located in the centre of the tubule. (**B**) The tubules of the dendritic ovary are embedded in the parenchyma **(P)**, which is surrounded by a thick layer of smooth muscle **(M)**. (**C**) The dendritic ovarian tubules are embedded in the parenchyma **(P)** and enveloped by a thick layer of smooth muscle **(M)**. Two adjacent ovarian tubules are observed where oogonia **(Og)** are found at the periphery, although apparently diminished. The oocytes **(Oc)** have a single central nucleus (Nu), and they occupy the central position of the tubules, where no alteration is shown. (**D**) The oviduct is embedded in the parenchyma **(P)**, which is surrounded by a thick layer of smooth muscle **(M)**. Oocytes are present in the centre, and oogonia are present at the periphery of the tubule.

The morphological features of the ovarian tubules in trematodes collected from goats that were administered various mimotope sequences were found to be similar to those observed in parasites from goats in the control group. Specifically, oogonia were observed at the periphery of the tubules, showing small basophilic nuclei and scanty cytoplasm, while the central region was occupied by primary oocytes that presented a polyhedral shape with a small central nucleus and large cytoplasm. However, it should be noted that in certain instances, the tubules were not entirely filled with these oocytes (Figure 3). Hence, not all the trematodes that were collected had the pathology described by Hanna [31].

#### 2.3.3. Vitellaria of Parasitic Trematode *F. hepatica*

**Figure 4:**
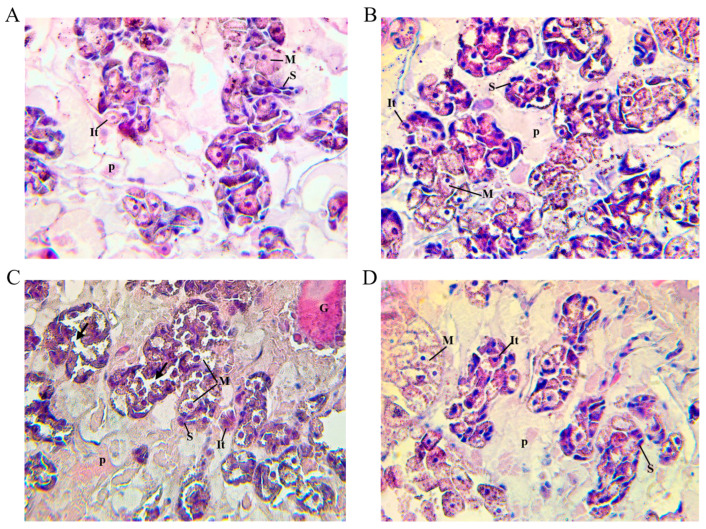
Light micrograph at 400× magnification of *Fasciola hepatica* yolk follicle tissue stained by H&E technique of vaccinated goats from group 1 (**A**), group 2 (**B**), group 3 (**C**), and unvaccinated control group 4 (**D**). (**A**) Within each follicle, maternal yolk cells **(S)** are present in a peripheral position. Intermediate vitelline cells **(It) are present**, while mature vitelline cells **(M)** are displaced towards the centre of the follicle. **P** = parenchyma. (**B**) Within each follicle, stem cells **(S)** and intermediate cells **(It)** are regularly observed, while more mature cells **(M)** are also identified. **P** = parenchyma. (**C**) Within the follicles, there are gaps (arrows) between the cells in the follicles. Stem cells **(S)** and intermediate cells **(It)** are present in smaller numbers than in group 4, and mature cells are irregular in shape. **P** = parenchyma; **G** = gut. (**D**) Yolk follicles with a high number of mature cells **(M)** as well as stem cells **(S)** and intermediate cells **(It)**. **P** = parenchyma.

Histological examination of the trematodes recovered from the twenty-four goats revealed cells in different stages of maturity in the yolk follicles, as shown by [30,31,32]. The follicles contained stem yolk cells with nuclei exhibiting heterochromatin and cytoplasm displaying basophilia, located at the periphery. Additionally, intermediate yolk cells were identified by the presence of protein globules in the cytoplasm. Mature yolk cells exhibited a significant concentration of protein globules displaced towards the centre of the follicle (Figure 4A–C). However, group 3, which received the cathepsin L1 SGTFLFS vaccination, exhibited cellular abnormalities. These abnormalities included indistinct cell borders, misalignment of globules around the cell periphery, gaps between certain yolk cells, and in some vacuoles, there were protein globules (Figure 4C). 

#### 2.3.4. Fasciola Hepatica Mehlis’ Glands

**Figure 5:**
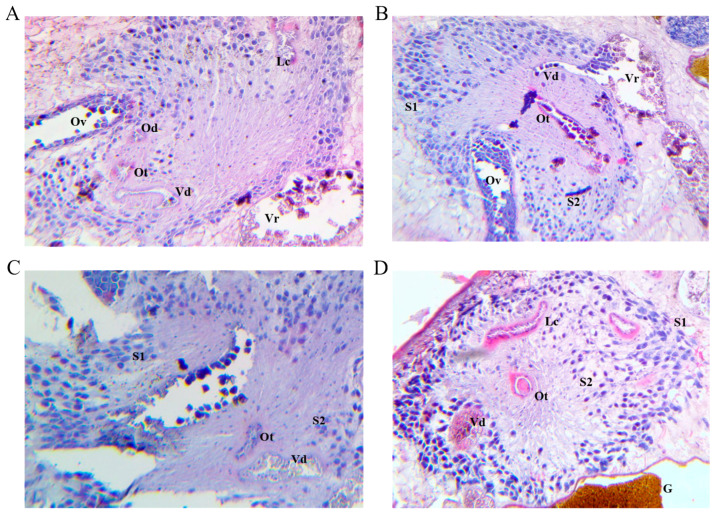
Light micrograph at 400× magnification of a histological section of *Fasciola hepatica* Mehlis’ gland tissue stained by H&E technique of vaccinated goats from group 1 (**A**), group 2 (**B**), group 3 (**C**), and unvaccinated control group 4 (**D**). (**A**) Ootype **(Ot)**, oviduct **(Od)**, ovary **(Ov)**, vitelline duct **(Vd)**, vitelline reservoir **(Vr),** and Laurer’s canal **(Lc)**. (**B**) Ootype **(Ot)**, ovary **(Ov)**, vitelline duct **(Vd)**, vitelline reservoir **(Vr)**, Mehlis’ gland types **S1** and **S2** cells. (**C**) Ootype **(Ot)**, vitelline duct **(Vd)**, Mehlis’ gland types **S1** and **S2** cells. (**D**) Vitelline duct **(Vd)**, Mehlis’ gland types **S1** and **S2** cells, ootype **(Ot)**, Laurer’s canal **(Lc),** and gut **(G)**.

The general Mehlis’ gland morphology of the four groups (Figure 5) was similar to that reported in untreated *F. hepatica* trematodes [30,31]. The gland contained type 2 secretory cells with eosinophilic cytoplasm, which were less dense than type 1 cells. Type 1 cells were larger and located further away from the ootype. These cells presented cytoplasmic extensions leading around the ootype. It is thought that their secretions under alkaline conditions, due to their high viscosity, contribute to the formation and fusion of the proteinaceous shell around the vitelline cells inside the ootype [22]. This complex is considered to be the trematode’s egg production chamber, where the oocyte and 30 vitelline cells fuse, and the egg develops.

#### 2.3.5. Uterine Contents of Liver Flukes

**Figure 6:**
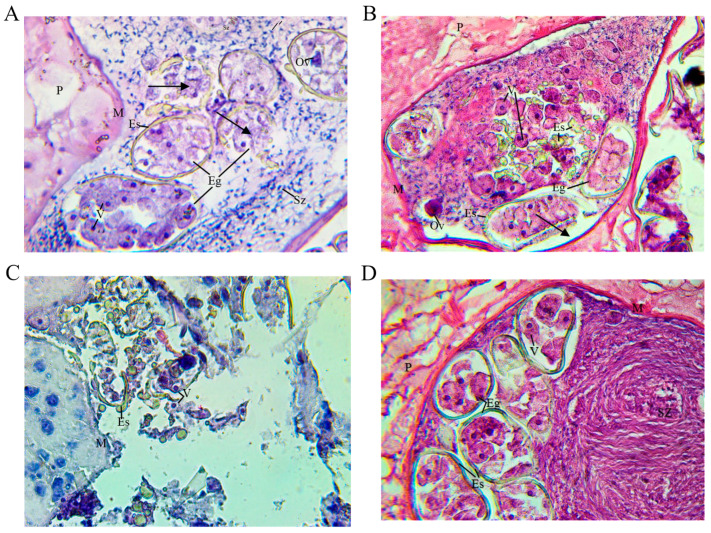
Light micrograph at 400× magnification of a histological section of *Fasciola hepatica* uterine tissue stained by H&E technique of vaccinated goats from group 1 (**A**), group 2 (**B**), group 3 (**C**), and unvaccinated control group 4 (**D**). (**A**) The uterine tubes are lined by smooth muscle (M) and surrounded by parenchyma **(P)**. Fully formed oocytes **(Eg)** and other irregular oocytes (arrows) are seen, each surrounded by a yellowish protein shell **(Es)** with approximately 30 yolk cells **(V)** and one oocyte containing an egg **(Ov)**. Numerous mature spermatozoa **(Sz)** are present in the uterine tube. (**B**) Some eggs **(Eg)** are perfectly encapsulated, whereas others are fragmented, showing free vitelline cells **(V)** in the uterus and accumulations of yellowish protein shells **(Es)**. The presence of an ovum **(Ov)** and mature spermatozoa **(Sz)** can be seen. Also note the empty space at the periphery of the fallopian tube, indicating reduced semen content. (**C**) There are no intact oocytes in the uterine tube, and there is a decrease in and destruction of vitelline cells **(V),** and there are some clumps of yellowish protein shell **(Es)**. No spermatozoa are present. (**D**) Defragmentation of the yellow protein envelope **(Es)** is observed. Some yolk cells inside the decapsulated protein shell are seen free in the uterus, showing a small number of cells without nuclei. Mature spermatozoa **(Sz)** and a single ovum **(Ov)** are present in smaller numbers.

The eggs observed in the proximal uteri of the trematode control group were fully formed and enveloped by proteinaceous shell material, as reported by [30,31]. In the samples obtained from vaccinated goats in groups 1 and 2, we detected fully formed eggs with yolk cells enveloped by a proteinaceous shell, but not all eggs contained ova (Figure 6). We also observed some irregular eggs with a few fragments or accumulations of proteinaceous shell material and some free yolk cells. However, in group 3, no fully formed eggs were observed in the proximal uterine tissue, and there was a greater accumulation of proteinaceous shell fragments. Another finding was the alteration of some yolk cells, which were anucleate.

## 3. Discussion

A parameter commonly used to assess the efficacy of vaccines is the reduction in the number of flukes and faeces eggs, which helps to decrease the dissemination of eggs and contamination of pastures, and, consequently, the prevalence of *Fasciola* infection in regions where the disease is prevalent [33]. The findings of this investigation demonstrated that immunisation using phage clones of cathepsin L2 (group 1) and cathepsin L1 (group 2 and group 3) combined with Quil A adjuvant resulted in a significant reduction in fluke burden, with reductions of 50.4%, 62.2%, and 75.3%, respectively. Our results were similar to previous research conducted on goats, wherein we found a reduction in fluke burden ranging from 55.40% to 79.53% using a 7-mer peptide disulphide-constrained approach [33,34]. Furthermore, the aforementioned findings align with previous studies conducted on mice (45.83–66.67%; 12-mer peptides) [35], and sheep (33.91–57.58%; 7-mer peptide disulphide-constrained and 12-mer peptides) [33,34,36]. These studies utilised different random peptide phage display libraries and challenged them with metacercariae of *F. hepatica*. The excretory/secretory (E/S) products contain cathepsins L (CLs) as the predominant constituents, which play crucial roles in various biological processes, including immunological regulation, tissue invasion, nutrition acquisition, and egg production [16,37]. The mean percentage of fluke implantation (47.6%) in the unimmunised control group was within the reported range of prior experimental *F. hepatica* infections in goats, which exhibited implantation rates ranging from 42.6% to 46.0% [19,20,33]. The present investigation observed a noteworthy reduction in fluke burden, concomitant with a substantial decrease in faecal egg count (FEC) for group 1 (PPIRNG, 52.0%), group 2 (DPWWLKQ, 47.5%), and group 3 (SGTFLFS, 71.0%). These outcomes align with the results reported by [33], where goats were vaccinated with the same sequences of filamentous phage peptide libraries. However, other trials have indicated that the use of recombinant cathepsin L1 with Quil A did not result in statistically significant reductions in the faecal egg count (FEC) [19,20].

The fluke burden of goats vaccinated with cathepsin L1 (group 2: DPWWLKQ) and cathepsin L1 (group 3: SGTFLFS) was significantly correlated with total IgG, reflecting the importance of antibodies in protecting against parasite infection. However, goats vaccinated with cathepsin L1 (group 3: SGTFLFS) mimotopes provided the most significant protection (75.3%) and higher levels of IgG2 than those animals vaccinated with cathepsin L2 (group 1: PPIRNG, 50.4%), and low levels of antibodies. Ruminants infected naturally with *F. hepatica* metacercariae induced a highly polarised response towards IgG1 classes, indicative of a potent Th2-driven modulation of the host immune response and prolonged survival, residing in the liver or bile ducts [38,39]. The predominance of the Th2 immune response over Th1 is indicated by increased levels of cytokines such as IL-4, IL-5, and IL-10, and very little IFN-γ [40]. A high predominance of IgG2 over IgG1 antibodies was observed in animals vaccinated with phage clones of cathepsin L1, indicating that protection is associated with the induction of a Th1 or mixed Th1/Th2 response. This result is consistent with previous studies that demonstrated the potential efficacy of utilising phage clones of cathepsin L with Quil A for vaccinating goats and sheep [33,34]. In addition, analysis of cytokine levels in sheep vaccinated with cathepsin L mimotopes of *F. hepatica* showed higher IFN-γ levels and decreased IL-4 production [41].

The presence of cathepsin L has been identified through the use of immunohistochemistry in oocytes, as well as in the Mehlis’ and vitelline glands of *F. hepatica* [42]. The ootype acts as the anatomical location for the process of egg formation, which occurs at a rate of one egg every 3.46 s. This intricate process involves the release of a mature oocyte from the ovary, spermatozoa from the seminal receptacle, approximately 30 vitelline cells from the vitelline reservoir, and secretions from the Mehlis’ gland [22]. Previous work by our research group using Quil A adjuvant in goats [33] showed a significant reduction in the total area of reproductive organs. Additional experiments also showed that the immune response in vaccinated mice specifically targets the Mehlis’ gland cathepsin L, leading to interference in eggshell synthesis [22]. Furthermore, animals that received CL mimotopes exhibited a significant reduction in the total area of their reproductive structures.

In haematoxylin and eosin-stained sections of seminiferous tubules that were treated with anthelmintics such as triclabendazole, compound alpha, and ivermectin [30,43,44], a progressive and marked increase in eight-cell rosettes of primary spermatocytes with degenerative features showing condensed or fragmented multiple nuclei, pyknotic nuclei, and eosinophilic cytoplasm was observed (eosinophilic bodies), indicating that they were undergoing apoptosis [21,29,43], as well as moderate peripheral vacuolation and fewer primary and secondary spermatogonia [29]. These cellular changes were found in trematodes from groups 1–3 in the present study but were more prevalent in group 3. The observation revealed the presence of a moderate quantity of cells exhibiting degenerative characteristics, including numerous nuclei, pyknotic or karyorrhectic nuclei, and eosinophilic cytoplasm, possibly consistent with apoptosis [45], but diagnostic tests to confirm this type of cell death are lacking. The degenerating vitelline follicles observed in group 3 were consistent with other studies [23,30,46] using triclabendazole and compound alpha. It should be noted that in these results, the conditions are reported in smaller numbers.

The main finding of this study was that, in the irregular production of *F. hepatica* eggs using different mimotope sequences, a marked alteration of normal egg formation was observed in histological sections of parasites collected from goats vaccinated with the phage clone SGTFLF, with more moderate effects than those found in studies by [23,29,30,32] using the anthelmintics triclabendazole and compound alpha as a treatment. The alteration of egg formation and structure found in our study does not fully clarify which of the tissues involved in this highly complex mechanism is not functioning properly. We suggest that the coordination of events in the oocyte and proximal uterus may have been disrupted: i.e., the secretion of meibomian gland cells, the mixing of yolk cells with the release of proteinaceous material, the formation of the eggshell, and the shaping of the egg [32].

## 4. Materials and Methods

### 4.1. Vaccination and Experimental Infection

**Figure 7:**
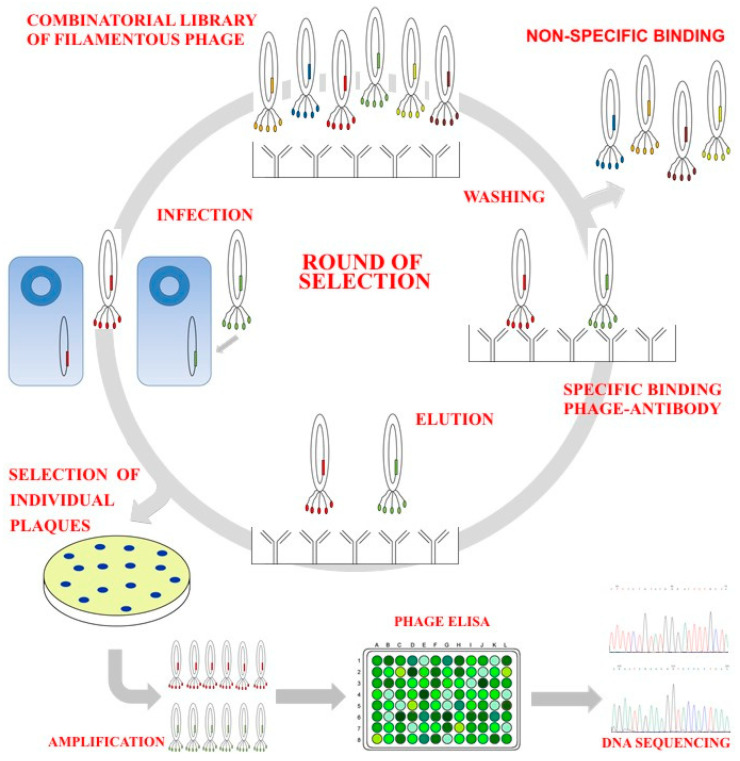
In vitro phage biopanning. A phage display peptide library with a complexity of billions of phage clones is selected via affinity with anti-cathepsin IgG. After incubation, the unbound phages are washed, the bound phages are eluted, amplified, used to infect *E. coli* strain ER2738, and titrated for the next round of screening. After three rounds, the affinitive phages are enriched, and individual plaques are picked up randomly, and identified by DNA sequencing.

A total of twenty-four six-month-old goats from a disease-free production system were selected for this study. We performed a thorough examination on the goats to determine the absence of *F. hepatica*. This examination involved analysing their faeces and testing their serum antibodies using an indirect ELISA test as described by [34], using excretion/secretion products of the parasite as an antigen. A cohort of 24 goats was divided into four groups according to body weight, with each group consisting of six animals. Each group was housed in a separate pen with a concrete floor and water ad libitum. The control group received an injection of sterile phosphate-buffered saline (PBS), while groups 1–3 were immunised subcutaneously with 1 × 10^13^ phage particles containing the sequences PPIRNGK (cathepsin L2), DPWWLKQ (cathepsin L1), and SGTFLFS (cathepsin L1). These phage particles were selected from previous studies (Figure 7) using rabbit polyclonal IgG antibodies against cathepsin L detected by Western blot [34,36]. The immunisation was performed in combination with 1 mg/mL Quil A adjuvant in sterile PBS at a pH of 7.2 (Accurate Chemical & Scientific Corp., Westbury, NY, USA). Every goat was administered the initial subcutaneous injection at week 0, followed by a second injection at week 4. The eggs of *F. hepatica*, extracted from bovine livers obtained from a slaughterhouse in Atlixco Puebla, were incubated with tap water in complete darkness at 22 °C for 14 days. The eggs were examined microscopically for evidence of miracidium development and exposed to the light of a 100 W lamp for 15 min to stimulate miracidia hatching. Laboratory colonies of *Lymnaea cubensis* snails were used and snails were kept individually in a flat-bottomed micro-ELISA plate with 0.35 mL/well of PBS and 3 miracidia/snail for 4 h at 22 °C. Subsequently, the snails were transferred to petri dishes on mud supplied with the blue-green alga *Oscillatoria* sp. The infected snails released *F. hepatica* cercariae after light stimulation and were counted under a stereoscopic microscope [47]. On the sixth week of the study, all goats were subjected to a single administration of 200 metacercariae, which were less than two months old, and the viability was confirmed by microscopy prior to the infection. The metacercariae were supplied orally to the goats using gelatine capsules and oesophageal forceps.

### 4.2. Evaluation of Vaccination Efficacy

All goats were humanely euthanised by exsanguination following captive bolt stunning 22 weeks after the first immunisation (week 0). Subsequently, the flukes present in the main bile ducts and gall bladder were retrieved and quantified. The faecal samples were individually obtained from the rectum manually, utilising disposable plastic examination gloves. Afterwards, they were placed in a plastic bag, appropriately marked, and promptly transported to the laboratory while maintaining refrigeration. The samples were processed within a maximum of 12 h after collection. Duplicate analyses were conducted on five grammes of each sample using the sedimentation technique (week 22). The resulting egg counts were represented as the number of eggs per gramme of faeces (EPG) ± standard deviation.

### 4.3. Humoral Responses Induced by Vaccination

Blood samples were collected by jugular venipunctures into vacutainer tubes (Becton Dickinson, Franklin Lakes, NJ, USA) before the first immunisation with phage clones at two-week intervals until the end of the experiment at week 22. Blood was allowed to clot, and serum was stored at −80 °C until use. The levels of anti-*F. hepatica* IgGs were determined using an indirect ELISA as described by Villa-Mancera et al. [41], with minor modifications. Briefly, a 96-well microtiter plate (Costar, Corning, NY, USA) was coated with phage clones of CL1 or CL2 mimotopes (1 × 10^10^ pfu) in 100 μL of PBS and incubated overnight at 4 °C with gentle shaking. After each incubation step, the microtiter plates were washed five times with PBS containing 0.05% Tween 20 (PBS-T). The plates were blocked with 200 μL of blocking solution (1% bovine serum albumin in PBS) for 1 h at 37 °C and subsequently incubated with 100 μL of serum diluted to 1:100 in PBS at 37 °C for 1 h. The wells of microtiter plates were incubated with HRP-conjugated donkey anti-goat IgG (1:5000, Jackson Immuno Research, West Grove, PA, USA), human anti-bovine IgG2 isotype (HCA020A, 1:500, AbD Serotec, Oxford, UK), or biotinylated sheep anti-bovine IgG1 isotype (ab106510, 1:10,000, Abcam, Cambridge, MA, USA) as secondary antibodies to estimate the Th1/Th2 balance, and were added in blocking solution for 1 h at 37 °C. Reactivity was detected by the addition of 3,3,5,5-tetramethylbenzidine (TMB; Sigma-Aldrich, St. Louis, MO, USA) substrate, stopped with 50 μL of 4 N H_2_SO_4_, and the absorbance of triplicates from each serum sample was read at 450 nm using an ELISA reader (Biotek ELx800, Winooski, VT, USA). Pre-immune goat serum, which does not react with *F. hepatica* cathepsin L proteinase, was used as a negative control.

### 4.4. Preparation of Trematodes and Histological Sections

At least ten *F. hepatica* of goats were fixed in a 10% (*v*/*v*) solution of neutral buffered formalin for a duration of 24 h. The selection of the sample size for histology was based on the quantity of flukes obtained from each individual goat. Furthermore, the lack of significant histological variation was noted among vaccinated groups 1–3 and the unvaccinated control group 4, which significantly contributed to their influence. The trematodes were compressed between two glass slides, and, following a 24-h period of fixation, the slides were then removed. Conventional techniques were employed to perform histological processing and paraffin embedding [29]. The process of dissecting each adult parasite that had been preserved in formalin, aligning them in wax-embedding, and subsequently cutting them from the wax blocks was carried out following the methodology described by [21]. Thin sections measuring 5 µm in thickness were obtained from both sides of the wax block. The cut surfaces were presented to face the block face and thereafter subjected to staining with haematoxylin and eosin (H&E) using standard histological procedures [48]. The sections were analysed, and images of the reproductive tissues were photographed using a calibrated Axioskop 2 plus microscope (Carl Zeiss, AG, Jena, Germany) equipped with an AmScope MU800 digital camera (Amscope Inc., Irvine, CA, USA). The number of primary and secondary spermatogonia were counted in 8 randomly selected transversally sectioned seminiferous tubules per animal. Results were given as mean ± SD per group. 

### 4.5. Statistical Methods

Values for liver fluke counts are presented as mean ± standard deviation. Percent reduction of parasite burden was calculated as (mean of control group − mean of vaccinated group)/mean of control group × 100. Data were analysed with IBM SPSS Statistics version 25 for Windows (SPSS Inc., Chicago, IL, USA), and the significance level was set to 0.05. The Kolmogorov–Smirnov test was performed to confirm the normality of the distributions. The comparison of results between groups was carried out using Student’s t-test when distributions of the data were normal, and a Mann–Whitney U test where the distributions were nonparametric. Correlations were calculated with Spearman’s non-parametric correlation test to assess the relationship among total IgG, IgG1, and IgG2 in the serum and fluke burden.

## 5. Conclusions

The findings of this study provide evidence that vaccination with cathepsin L mimotopes in combination with Quil A adjuvant to goats resulted in significant reductions in liver fluke burden and faecal egg count. Immunisation elicited high levels of both IgG1- and IgG2-specific antibodies, which provided significant protection. Further trials involving a larger number of animals emulsified with different adjuvants are required to ensure the efficacy of vaccination. In addition, parasites obtained from the immunised groups were unable to develop normal eggs, indicating a failure of the highly complex mechanism of egg formation in utero. Further studies on the use of filamentous phage clones are warranted, using vaccine formulations containing newly discovered antigens of juvenile and adult flukes to enhance the effectiveness of *F. hepatica* vaccines and mitigate the prevalence of ruminant fasciolosis in regions where the disease is prevalent.

## Figures and Tables

**Table 1:** Reduction in fluke burden and faecal egg counts of vaccinated goats using cathepsin L1 and L2 mimotopes and an unvaccinated control group after challenge with 200 *Fasciola hepatica* metacercariae at week 6.

	Group 1	Group 2	Group 3	Group 4
	**Vaccination with mimotopes:**			**Control (PBS)**
	CL2: PPIRNG	CL1: DPWWLKQ	CL1: SGTFLFS	
**Flukes recovered**				
Individual data	39, 71, 57, 29, 24, 63	31, 39, 33, 28, 42, 43	24, 13, 26, 16, 32, 30	63, 74, 106, 119, 112, 97
Mean ± SD	47.2 ± 19.2	36.0 ± 6.2	23.5 ± 7.6	95.2 ± 22.2
Reduction rate (%)	50.4 *	62.2 *	75.3 *	-
**Faecal egg counts**	58.6 ± 37.5	64.0 ± 25.4	35.4 ± 14.5	122.0 ± 44.0
**Reduction in FEC (%)**	52.0 *	47.5 *	71.0 *	-

FEC: faecal egg counts; CL: cathepsin L. Significant differences with the control group (*p* < 0.05) are indicated by a single asterisk (*).

## Data Availability

The data presented in this study are available on request from the corresponding author.
